# Report of Part of My Surgical Work for the Six Months Ending April 15th, with Remarks

**Published:** 1895-05

**Authors:** J. B. S. Holmes

**Affiliations:** Atlanta, Ga.


ATLANTA
Medical and Surgical Journal.

VOL. XII. MAY, 1895. No. 3.

LUTHER B. GRANDY, M.D., MANAGING EDITOR.

MILLER B. HUTCHINS, M.D., BUSINESS MANAGER.

### ORIGINAL COMMUNICATIONS

### REPORT OF PART OF MY SURGICAL WORK FOR THE SIX MONTHS ENDING APRIL 15TH, WITH REMARKS.*

BY J. B. S. HOLMES, M.D.,
ATLANTA, GA.

I herewith beg to submit a condensed report of some of my surgical work for the past six months, all done in private practice, and most of it in my sanatorium.

Many of the cases are commonplace and devoid of any special interest, while others are quite worthy of your attention. In no case has an ovariotomy been performed simply for the relief of nervous or reflex symptoms. I have operated only for disease; a condition that I knew could only be relieved by surgical procedure. Careful examination, post-operative, has confirmed my opinion in every instance.

While I believe in conservative measures, the most radical are often the most conservative. I am ready to admit that the abdominal cavity has often been ruthlessly invaded and organs removed that should not have been touched. They have been removed for nervous symptoms due entirely to other causes, and for which they were in nowise responsible. On the other hand, tinkering gyne-

*Read at the meeting of the Georgia Medical Association, Savannah, April 19, 1895.

cology, I am sure, is responsible for many diseased tubes and ovaries that can only be cured by removal. The introduction of various caustic remedies into the uterus, causing destruction and sloughing of the mucous membrane, with a cervical canal in many cases previously constricted, or so rendered by the applications that the decomposing masses cannot pass out. What follows? A septic endometritis, which soon extends to the tubes and ovaries, producing trouble that nothing but the knife will relieve. This must be resorted to, or the poor woman is frequently doomed to permanent invalidism—a condition sometimes worse than death. Many of the cases that the surgeon is censured for using the knife upon are caused by such treatment. I do not condemn all local treatment. Properly directed, I believe there are many conditions where it is not only admissible, but demanded. Often in these conditions cures are made, especially where the local treatment is supplemented by constitutional measures, for oftentimes local troubles are clearly the result of constitutional vices. I do condemn, however, in the strongest terms, the reckless, haphazard, and indiscriminate introduction of the various caustics into the uterus; certainly none should be introduced without first providing for thorough drainage. The want of cleanliness on the part of many physicians when making vaginal and uterine examinations is often a potent factor in causing uterine and pelvic disease. Not only should the hands and instruments be clean, but the vagina and cervix should be thoroughly cleansed and disinfected before an instrument of any kind is passed into the uterus. How many of you have seen your professional brother swab the uterus with all sorts of medicaments, using dirty cotton on an equally unclean applicator, never cleansing the vagina and cervix, and even passing a sound into the uterus that had been passed in perhaps a dozen others without coming in contact even with soap and water. While the uterine sound is a valuable instrument in its place, there are but few instances where it is required. I believe it has done more damage to women than all other instruments combined, and it not only has been but is still too indiscriminately used. Stem pessaries have made many victims for the surgeon's knife. With the possible exception of the uterine sound, they have done more harm than any other instrument or appliance.

Understand me, I do not think that every diseased ovary should be removed; far from it. I decline operation in many cases where the patient is anxious for it. I try to be conscientious and operate only where the disease is so advanced that it has or is likely to render the woman an invalid, and is incurable by any other means.

CASE 1.—Miss M., aged thirty-eight, Georgia. Noticed a small lump in the left mamma in the spring of 1894. This gradually grew, and I saw her in the fall of the same year. The darting pains through the mamma and left side of the chest, at times, were quite severe. When examined, the tumor was about the size of a hen's egg, extremely hard and quite sensitive. No involvement of the axillary glands; her general health not good; no history of cancer in the family. After purging her well, the skin over the gland was thoroughly cleansed with oil of turpentine, then tincture of green soap, then with a bichloride solution, one to one thousand. An elliptical incision, in the direction of the fibers of the pectoralis major muscle, was made, and the entire gland removed, leaving bare the muscular tissue underneath. The wound was thoroughly cleansed with boiled water and made dry with sterilized gauze. A rubber drainage-tube was laid in the bottom of the wound, extending its entire length. The edges were brought well together, silkworm gut being used as sutures. A dressing of sterilized gauze and absorbent cotton was applied over the incision and held in place with adhesive straps, and then bandages around the chest. At the end of forty-eight hours the drainage-tube was removed, when perfect union in the entire length of the incision was found. She has had no indications of a return of the disease, is free from pain, and her general health much improved.

CASE 2.—Miss E., aged thirty-five, Alabama. Came under my observation in October, 1894, suffering with intense headache, chiefly in the occipital region, insomnia, severe indigestion, extreme nervousness, frequent and painful micturition, intense backache, inability to walk, severe pain in the right iliac region, greatly increased when riding or standing, pain frequently of a throbbing character; menstruation regular, but intensely painful for twentyfour hours before and twenty-four to thirty-six hours after the flow commenced. Upon examination, I found the right kidney loose, the right ovary prolapsed, enlarged, extremely sensitive, and firmly fixed by inflammatory adhesions in Douglas's sac; the left also prolapsed, considerably enlarged, and quite sensitive. On October 18th, after the usual preparation, both ovaries and tubes were removed. My diagnosis was confirmed in every particular. Both ovaries were found filled with numerous cysts, and almost the entire stroma destroyed. She left the sanatorium December 1 free from pain, digestion much better, sleeping well, and nervousness greatly improved. She has continued to improve, as recent letters have indicated. She was, however, advised that before her nervousness entirely disappeared it might be necessary to anchor the loose kidney. This I shall do during the coming summer, unless she is so comfortable as to render the operation unnecessary.

Case 3.—Mrs. C., nullipara, Kentucky, married two years. Began to menstruate at fifteen without pain until marriage. Up to marriage had been in excellent health, weighing one hundred and forty-three pounds. Soon after marriage suffered an attack of pelvic peritonitis, and from that time until she came under my observation her health had steadily given way, and when I saw her she only weighed ninety pounds. Suffered with insomnia, no appetite, poor digestion, constipation, frequent and painful micturition, backache, intense pain in the pelvis, most severe on the left side; constant bearing down sensation while standing or walking. On examination found the uterus firmly fixed, the left ovary and tube very much enlarged, also fixed, extremely sensitive and tender. The right also fixed and enlarged, but not to the same extent. An ovariotomy was done October 20th, and upon opening the abdomen I found the ovaries and tubes adherent to everything within their reach. After much difficulty the adhesions were broken up and the ovaries and tubes removed. The abdomen was thoroughly irrigated with boiled water at a temperature of one hundred and five and glass drainage used, the tube remaining forty-two hours. Her recovery was uninterrupted, and she is to-day in perfect health; free from pain, good appetite, perfect digestion, sleeps well, and weighs one hundred and twenty-five pounds. The left tube and ovary were both filled with pus, the right tube filled with blood and the right ovary cystic.

CASE 4.—Captain D., aged fifty-six, Alabama, came to my sanatorium October 20th, intensely weak, pulse one hundred and thirty, temperature one hundred and two, swelling in right iliac region as large as a fetal head. He stated that two weeks before he was seized with a severe pain three inches to the right and a little below the umbilicus, accompanied with high fever, constipation, and vomiting. He was treated for liver disease until he became so alarmed about his condition that, as ill as he was, he left home and came to Atlanta, consulting my friend, Dr. E. H. Richardson, who promptly referred him to me. Upon examination I diagnosed perforative appendicitis with suppuration. His condition was so extreme that I operated within an hour after he came into the sanatorium. He was given a hypodermic of strychnia, one-thirtieth of a grain, the skin over the abdomen was cleansed thoroughly with oil of turpentine, green soap, and then a bichloride solution, one to one thousand. An incision was made from McBurney's point, extending two inches downward. Just as my knife entered the abscess sack, he coughed, and the pus spurted at least three feet high. About a quart of extremely fetid pus was discharged. The cavity was thoroughly irrigated with boiled water, after which peroxide of hydrogen was freely used and the cavity packed with iodoform gauze. I did not search for the appendix, which I am sure had been entirely destroyed by suppurative process. The dressings were removed each day and peroxide of hydrogen used and the packing of iodoform gauze reintroduced. His improvement was continuous, and he left the sanatorium November 19th, with the wound entirely healed and he in excellent condition. His health has continued good, as letter of April 11th states.

CASE 5.—October 28, 1894, I was called to a neighboring village, twenty miles distant, to see Mrs. D., aged fifty-six, multipara. Upon my arrival I found that two weeks before she was seized with severe pain in the right iliac region, followed by considerable fever, accompanied with sick stomach. She presented the usual symptoms of perforated appendicitis. At the time I saw her a distinct tumor could be felt in the right iliac region. She had a temperature of one hundred and two and a half, pulse one hundred and twenty-five. It was then six o'clock in the evening, but her condition was so grave that I decided to operate immediately, and did so by lamplight. The incision was made just below McBurney's point, and about twelve to fifteen ounces of pus evacuated. The cavity was thoroughly irrigated with boiled water and then peroxide of hydrogen freely used; after which the cavity was packed with iodoform gauze, and a dressing of absorbent cotton applied. The gauze was removed each day, peroxide freely used, and the gauze reintroduced. I did not see her again, but her recovery was uneventful. A letter received from her four weeks after the operation stated that she was then perfectly well.

CASE 6.—Mrs. H., aged twenty-three, nullipara, Florida. Menstruation began at seventeen and continued without pain up to two years before consulting me, which she did in October, 1894. She was married nine months before I saw her, since which time her menstrual pain had been greatly increased. She gave a clear history of pelvic peritonitis occurring soon after marriage, Pain in abdomen most intense on right side; headaches, severe backache, inability to stand or walk with any comfort, constipation. Upon examination found right ovary enlarged, prolapsed, firmly adherent, and acutely sensitive; left ovary enlarged and firmly fixed in Douglas's sac. Both ovaries and tubes were removed November 22. The adhesions were easily broken up, as they were of recent standing and no drainage used. Both ovaries were filled with fluid and both tubes entirely occluded. She left the sanatorium December 25th, free from pain. Has steadily increased in weight, and at this time is entirely well.

CASE 7.—Mrs. H., aged thirty-two, multipara, North Carolina. Menstruation profuse and intensely painful, severe headaches, insomnia, acute pain in the back, bearing down sensation in pelvis, inability to stand or walk. Had not walked a step in nine months. A confirmed opium habitué. Upon examination I found the right ovary prolapsed and fixed in Douglas's cul-de-sac, and I think the most sensitive of any I have ever touched. The left ovary was prolapsed and cystic, about the size of a hen's egg: uterus retroflexed and considerably enlarged. On November 22d I removed both ovaries and tubes, elevating the uterus by shortening the round ligaments which were included in the ligatures encircling the ovarian pedicle. She left the sanatorium December 24th free from pain and walking without discomfort. She is now attending to her household duties and in perfect health. Was entirely free from the opium habit before leaving the sanatorium and has so continued.

CASE 8.—Mrs. G., aged thirty-four, primipara, Alabama. Headache, constipation, frequent and painful urination, very nervous, pain in the abdomen, shortness of breath, palpitation of the heart, intense backache, greatly increased since second marriage six months ago. Upon examination found right ovary fixed, enlarged, and very tender; left in about the same condition as right. Uterus large and immovable. An ovariotomy was done December 5th. The adhesions were very dense and numerous. The ovaries and tubes were enucleated with great difficulty. The abdomen was thoroughly irrigated with boiled water, and a glass tube inserted, the tube remaining twenty-six hours. She returned home January 15, 1895, entirely free from pain, and at last advices was doing well in every particular.

CASE 9.—Miss H., aged twenty, Georgia. Menstruation regular, but extremely painful, constant and intense backache, greatly increased when standing or walking; in fact, so severe as to make it almost impossible for her to walk or stand. Sharp pain low down in right side, frequently of a throbbing character, and always intensified when standing. Insomnia, frequent headaches, constipation, urination scant and painful, no appetite, poor digestion, quite anemic. Had spent most of the time for the past three years in bed. Right ovary enlarged to near the size of a hen's egg, and completely destroyed by cystic degeneration; left also cystic, prolapsed, and extremely sensitive. Ovariotomy done December 6th, removing both ovaries and tubes. Her convalescence was rather tedious, a mild phlebitis developing in the left leg at the end of the second week. This rendered her convalescence rather slow, and she left the sanatorium February 5th, improved, but not entirely relieved. Her improvement, however, since that time has been continuous. I saw her March 31st. She was able to walk wherever she pleased, ate and slept well, free from pain, had gained considerable in flesh, and expressed herself as being well and happy.

CASE 10.—Mrs. D., aged thirty-six, multipara, Florida. Menstruation regular but with great pain, violent headache, insomnia, no appetite, poor digestion, constipated, nausea, intense pain in back and lower abdomen, extending down the thighs; unable to walk, and had been confined to her bed for six months; an invalid for fifteen years. Had been treated locally much of the time for the past ten years. Found floating kidney on the right side, uterus enlarged and retroflexed, right ovary the size of a guinea's egg, firmly fixed in Douglas's sac; the left ovary prolapsed, cystic, sensitive. Ovariotomy performed December 18th, removing both ovaries and tubes and elevating uterus. Her convalescence from the operation was satisfactory in every particular. On February 5th the right kidney was anchored. I made an incision about two and one-half inches from the spine, beginning at the lower border of the twelfth rib and extending three and one-half inches in an oblique direction towards the crest of the ilium. When the kidney was exposed the capsule was incised and folded back one-fourth of an inch on each side. Four silkworm gut sutures were passed through the muscles, the fatty capsule of the kidney, and through the kidney tissue. Twelve strands of silkworm gut were placed on the kidney and brought out at the upper and lower angles of the wound. The kidney was pressed firmly in position by the hand of my assistant, then three additional sutures of silkworm gut were passed from the skin through the muscles, fatty capsule, and kidney tissue. The four deep sutures were drawn moderately tight and tied, cut close, and the cutaneous wound closed by the three deep sutures and several superficial ones. A dressing of sterilized gauze and absorbent cotton was applied over the wound, and a pad of gauze was applied over the kidney, on its anterior aspect, and held in position by rubber straps passing half round the body. On the eighth day the silkworm gut threads were removed and also the sutures. Perfect union had taken place throughout the entire length of the wound. The abdominal pad was worn for about thirty days. At this date, April 9th, the kidney remains firmly fixed, and the patient is walking about the sanatorium, and will return to her home on the 20th, comfortable and happy.

Case 11.—Mrs. H., aged thirty-five, nullipara, Georgia. Menstruation began at sixteen, always painful and very profuse, severe headache, no appetite, poor digestion, constipation, painful urination, and a feeling of constant pressure on the bladder. Suffered for three years with intense pain in abdomen, much greater in the region of the ovaries; very excitable, great mental disturbance. Had been treated locally for three years. On examination I found the uterus enlarged and fixed, the right ovary as large as a goose egg, tube very much enlarged, and both firmly fixed. The left ovary and tube also fixed, the tube considerably thickened and ovary cystic. Ovaries and tubes removed December 21st. Adhesions were very dense and strong, and appendages were removed with the greatest difficulty. The right ovary was ruptured in removing it and a large quantity of dark fluid discharged. The abdomen was thoroughly irrigated with boiled water, and glass drainage used, the tube remaining in twenty-four hours. The right tube, upon examination, was found filled with blood, the left ovary and tube filled with serum. Both tubes entirely occluded, and ovaries and tubes studded with papillomatous growths. Union of the abdominal incision in this case was interrupted by several stitch-hole abscesses, produced no doubt by the stitches being too tightly tied. She left the sanatorium January 23d, since which time she has continued to improve, and is now in full control of the care and management of a large hotel.

Case 12.—Mrs. N., aged thirty, nullipara, Georgia. Severe headache, no appetite, indigestion, constipation, frequent and painful urination, backache, pain in lower part of abdomen, increased by standing or walking. Pain in both iliac regions, but worse in the left. Painful menstruation, particularly so for twenty-four hours before the flow began. Very nervous and quite weak and anemic; some temperature in the afternoon. Upon examination, I found the right ovary prolapsed, tender, the tube very much thickened; left ovary prolapsed and firmly fixed to the side of the uterus and tube considerably enlarged. Ovaries and tubes removed January 5th. The right ovary contained a number of cysts. The left contained about a teaspoonful of pus, and both tubes were entirely occluded. She left the sanatorium February 11th, free from pain, with good appetite, good digestion, sleeping well, nervousness greatly relieved. She writes me about a month after going home that she walks where she pleases and suffers no pain when either walking or riding; that appetite and digestion have continued good, and she is entirely well in every particular. She had been under the care of several physicians and had had local treatment for many months.

CASE 13.—Mrs. M., aged twenty-eight, nullipara, Georgia. Intense headache, appetite variable, digestion very poor, bowels constipated, menstruation regular but intensely painful; severe backache, pain in lower part of abdomen, but much more severe on the right side. Had been bedridden for five years. Very weak and anemic. On examination found ovary very large, extremely sensitive, and about the size of a guinea's egg. Left tube and ovary both prolapsed and firmly fixed by inflammatory adhesions. She had had several well defined attacks of pelvic peritonitis. Both ovaries and tubes were removed January 5th. The adhesions were particularly dense and strong, especially on the left side. The abdomen was thoroughly irrigated with boiled water and glass drainage used, the tube remaining seventeen hours. Both ovaries were filled with a cheesy mass, evidently tubercular in character. Both tubes contained a small quantity of the same material. She is of tubercular family. She left the sanatorium March 22d strong enough to go where she pleased about the building, eating and sleeping well. Her improvement has been continuous and satisfactory since her return home. She had been treated locally for five years.

CASE 14.—Miss S., aged twenty-two, Georgia. Intense headache, insomnia, no appetite, wretched digestion, constipation, painful urination, pain for two or three hours after defecation very intense. Menstruation began at fourteen, with little pain for three years, after which, intensely painful for the next three years; since which time the flow has ceased altogether; profuse leucorrhea, severe abdominal pains, intense backache, extending down the limbs. Was extremely nervous, very anemic. Upon examination found a floating kidney on right side, position of womb normal, left ovary and tube in Douglas's pouch, firmly fixed; right ovary enlarged and very sensitive. Fissure in the anus. January 15th, both ovaries and tubes were removed, and the sphincter ani thoroughly stretched; fissure curetted, incised and touched with pure carbolic acid. Upon examination of the specimens, found both ovaries entirely destroyed by cystic degeneration and both tubes occluded. She left the sanatorium April 2d, considerably improved, but not well.

CASE 15.—Mrs. G., nullipara, aged twenty-three, South Carolina, married two years. In excellent health up to a few months after marriage, when she had an attack of pelvic peritonitis; since which time she had suffered with an intense burning pain in the top of the head, pain in the back and limbs and in right side, greatly increased by standing or walking. Appetite good, digestion poor, bowels constipated, very nervous. Upon examination found the right ovary enlarged, prolapsed and fixed; left also enlarged and exceedingly tender. Both ovaries and tubes were removed January 29th. The right was entirely destroyed by cystic degeneration, the left also septic; both tubes occluded. She left the sanatorium March 9th, in a satisfactory condition in every respect.

CASE 16.—Mrs. L., aged eighteen, nullipara, Georgia, three and a half months advanced in pregnancy. In excellent health up to a month before consulting me; since which time she had suffered most agonizing pain in the rectum, coming on shortly after defecation and lasting for hours. Upon examination I found a large and angry fissure. Notwithstanding her condition, I advised operation, as I felt assured that if she continued to suffer as she was doing, abortion would be the result. On February 1st, the sphincter ani was thoroughly divulsed, the fissure curetted, incised, and touched with carbolic acid, and a two-grain opium suppository inserted into the rectum. A drachm of Hayden's viburnum compound was given every six hours for the first two or three days, after which it was given two or three times in the twenty-four hours for about a week. At no time did she have the slightest uterine pain. She returned home February 14th, entirely relieved. I saw her on the 5th inst., when she told me that she had not had the slightest return of her trouble, and was as comfortable and as well as she could possibly be in her condition.

(*Concluded next month.*)

